# Supplementary material for: Adaptive tip-enhanced nano-spectroscopy
Source: Nat Commun. 2021 Jun 8;12:3465. doi: 10.1038/s41467-021-23818-1 (PMC8187726; doi:10.1038/s41467-021-23818-1)
Supplement: Supplementary file 1 — Supplementary Information [file 41467_2021_23818_MOESM1_ESM.pdf]

Supplementary Information: Adaptive tip-enhanced nano-spectroscopy

Dong Yun Lee,[1] Chulho Park,[2] Jinseong Choi,[1] Yeonjeong Koo,[1] Mingu Kang,[1] Mun Seok Jeong,[3,4] Markus B. Raschke,[5,6,7] and Kyoung-Duck Park[1]

[1]*Department of Physics, Ulsan National Institute of Science and Technology (UNIST), Ulsan 44919, Republic of Korea*

[2]*Department of Energy Science, Sungkyunkwan University (SKKU), Suwon 16419, Republic of Korea*

[3]*Department of Physics, Hanyang University, Seoul 04763, Republic of Korea*

[4]*Department of Energy Engineering, Hanyang University, Seoul 04763, Republic of Korea*

[5]*Department of Physics, University of Colorado, Boulder, CO 80309, USA*

[6]*Department of Chemistry, University of Colorado, Boulder, CO 80309, USA*

[7]*JILA, University of Colorado, Boulder, CO 80309, USA*

(Dated: April 9, 2021)

SUPPLEMENTARY NOTE 1. MECHANISM OF DYNAMIC SPP MODULATION

While our work demonstrates the ability to control the surface plasmon polariton (SPP) properties of Au tip for tip-enhanced nano-spectroscopy for the first time, there have been many efforts to manipulate the SPP characteristics, e.g., field amplitude, polarization, and spatial field distribution, in plasmonic nano-structures. Recently, dynamical SPP control for plasmonic metal hole array [1], slits [2, 3], and gratings [4, 5] was demonstrated. Since these SPP controls are based on the coherent conversion from excitation light to SPPs [6], many interesting ideas on SPP control could be achieved under systematic control of excitation light. Specifically, several studies reported that the SPP condition is strongly influenced by the space-variant polarization state of incident light [7–10], as well as by the spectral and spatial coherence of incident light [11]. Therefore, making the customized excitation light for plasmonic devices is a simple but effective approach to control the SPP characteristics of them.

In our case of tip-enhanced nano-spectroscopy, an incident laser beam $\mathbf{E}_{inc}$ is tightly focused at the apex of Au tip with a high NA objective lens. Due to the depolarization effect of the high NA objective lens, the optical field distribution of the focused beam in the vicinity of Au tip can be expressed with the angular spectrum method as follows [12]:

$$\mathbf{E}_{focus}(x, y, z) = \frac{ide^{-i\mathbf{k}d}}{2\pi} \iint \mathbf{E}_{inc}\Big(\frac{k_x}{k}, \frac{k_y}{k}\Big) e^{-i(k_x x + k_y y \pm k_z z)} \frac{1}{k_z} dk_x dk_y, \tag{1}$$

where d is the path length between the initial point at lens and the focal point, and the wave vector $\mathbf{k} = (k_x, k_y, k_z)$ is expressed in Cartesian coordinates. Based on this model, we can consider the focused beam as a superposition of plane waves with different wave vectors, i.e., the excitation light can be decomposed into the optical field components with various polarization angles with respect to the tip axis. Therefore, partial optical field components which have the polarization direction close to parallel with respect to the tip axis can be effectively coupled into surface plasmon. On the other hand, the other field components are lost, and thus the excitation rate for tip-enhanced photoluminescence or Raman is significantly low in conventional set-ups. To enhance the field localization, i.e., excitation rate, a radially polarized beam [13] is used, yet the coupling efficiency is still low and it can be only used for the bottom-illumination mode set-ups. In our approach, through

wavefront shaping of an excitation beam with a spatial light modulator, we dynamically manipulate the amplitude, phase, and polarization of the SPP customized for the tip shape. Therefore, this approach can significantly enhance the excitation rate for any TERS or TEPL modality, e.g., bottom-illumination, top-illumination, and side illumination modes.

SUPPLEMENTARY NOTE 2. OPTIMIZATION TARGET AND CONVERGENCE OF THE WAVEFRONT SHAPING ALGORITHM

In its simplest form, the optimization target we use is a spectrally integrated intensity of TEPL or TERS spectrum. Specifically, for a-TEPL of a WSe_2 monolayer, the optimization target is an integrated TEPL intensity in the range of 749 $\sim$ 753 nm. For a-TERS of a WSe_2 monolayer, the target is the spectrally integrated intensity of $A_{1g} + E^1_{2g}$ Raman peak. For a-TERS of BCB molecules, the target is the Raman peak of $\sim$583.5 cm^{-1}. The algorithm optimizes the optical phase of all the segments in the spatial phase mask sequentially to achieve the maximum intensity for the target signals.

To confirm the convergence, we repeat the algorithm three times continuously with the target signal. With several control experiments with different tips, we can find that the saturation enhancement is obtained and the optimization is converged after completing a single cycle of the algorithm (see Supplementary Figure 1 below).

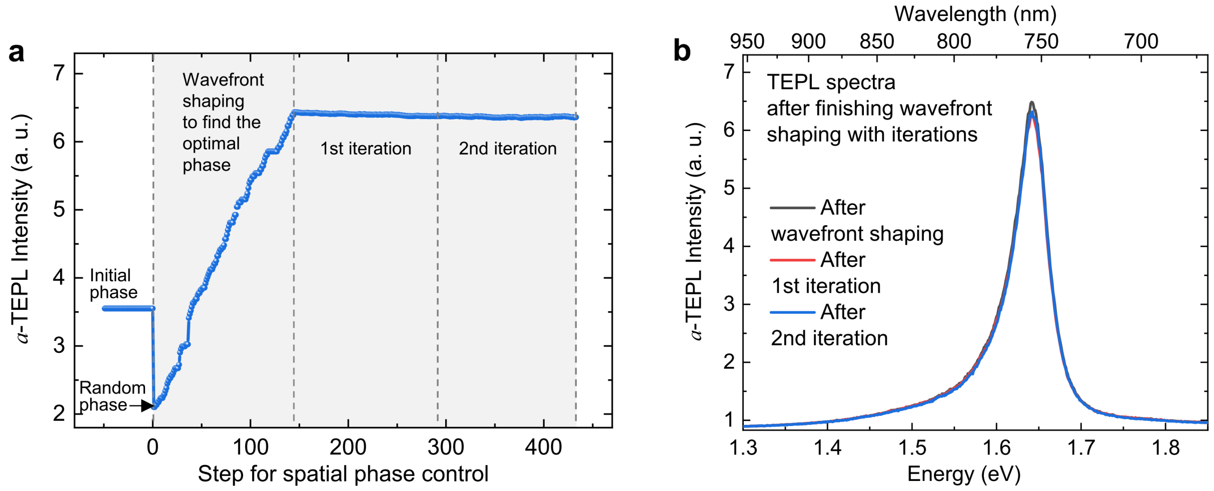

Supplementary Figure 1. (a) Evolution of a-TEPL intensity of a WSe_2 monolayer during the optimization of spatial phase mask by a stepwise sequential algorithm. The optimization algorithm is repeated 3 times to confirm the convergence. (b) a-TEPL spectra after finishing the wavefront shaping with iterations.

SUPPLEMENTARY NOTE 3. PHASE MASKS USED FOR *A*-TEPL AND *A*-TERS MEASUREMENTS

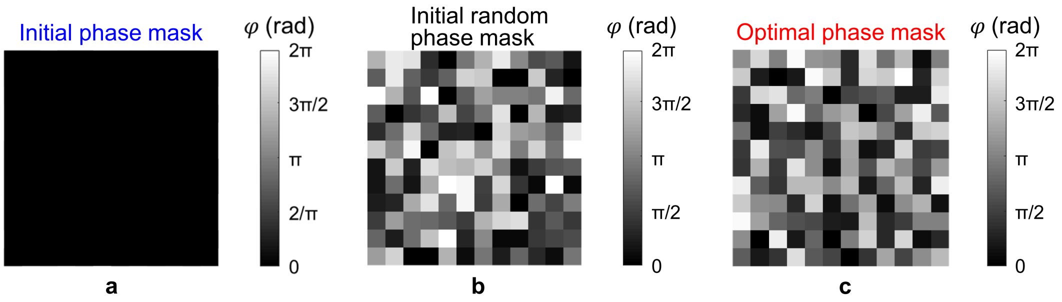

Supplementary Figure 2. The phase masks used for the wavefront shaping of the measurement Fig. 1b in the main text. (a) Initial phase mask, (b) initial random phase mask, and (c) optimal phase mask.

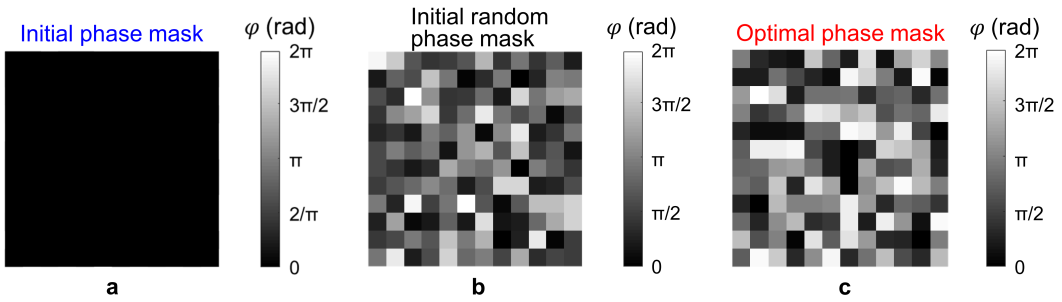

Supplementary Figure 3. The phase masks used for the wavefront shaping of the measurement Fig. 3a in the main text. (a) Initial phase mask, (b) initial random phase mask, and (c) optimal phase mask.

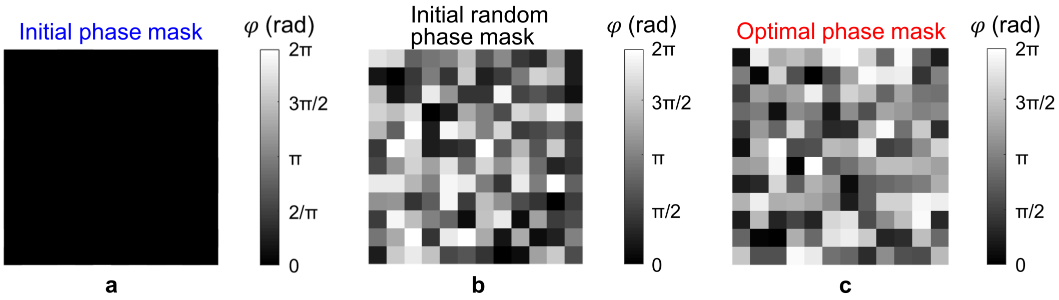

Supplementary Figure 4. The phase masks used for the wavefront shaping of the measurement Fig. 3e in the main text. (a) Initial phase mask, (b) initial random phase mask, and (c) optimal phase mask.

SUPPLEMENTARY NOTE 4. CALCULATION OF TEPL ENHANCEMENT FACTOR

For an atomically thin spontaneous emitter, i.e., WSe_2 monolayer, we can use the following equation for estimating TEPL enhancement factor (EF) [14]:

$$\mathrm{EF} = \left(\frac{I_{tip-in} - I_{tip-out}}{I_{tip-out}}\right) \times \frac{A_{FF}}{A_{NF}}, \tag{2}$$

where I_{tip-in} and $I_{tip-out}$ refer to the PL peak intensity with the tip approached (TEPL) and retracted (far-field PL) from the sample and A_{FF} and A_{NF} indicate the PL measurement areas corresponding to the focused beam spot by an objective lens and the near-field excitation region formed by Au tip, respectively. The values of I_{tip-in} and $I_{tip-out}$ are derived from curve fitting by a Lorentzian function. The values of A_{FF} and A_{NF} are derived using a general equation for an area of a circle πr^2 by considering a radius $r_{FF} \simeq (\lambda/2NA) \times 1.5 = 593.4$ nm (the empirical factor of 1.5 is used) [15] of the far-field laser spot and r_{NF} with half of the spatial resolution of the used Au tip, 7 nm (derived from Fig. 2c-d). Using Eq. (2) and the derived values from our experiment, we estimate the enhancement factor for the conventional TEPL without using the wavefront shaping as high as $\sim 1.9 \times 10^4$ (blue in Fig. 2a). By contrast, we achieve TEPL enhancement factor as high as $\sim 4.4 \times 10^4$ through the proposed wavefront shaping approach (red in Fig. 2a).

SUPPLEMENTARY NOTE 5. *A*-TEPL MEASUREMENT USING A RADIALLY POLARIZED BEAM

We also try *a*-TEPL experiment with a radially polarized excitation beam to confirm that the enhanced signal with the wavefront shaping is not just due to the radial polarization effect. As seen in Supplementary Figure 5 below, the wavefront shaping gives significant enhancement in *a*-TEPL intensity compared to the normal TEPL intensity, similar to the experiment with a linear polarizer. Therefore, the increased TEPL/TERS intensity using wavefront shaping is not just due to the radial polarization effect.

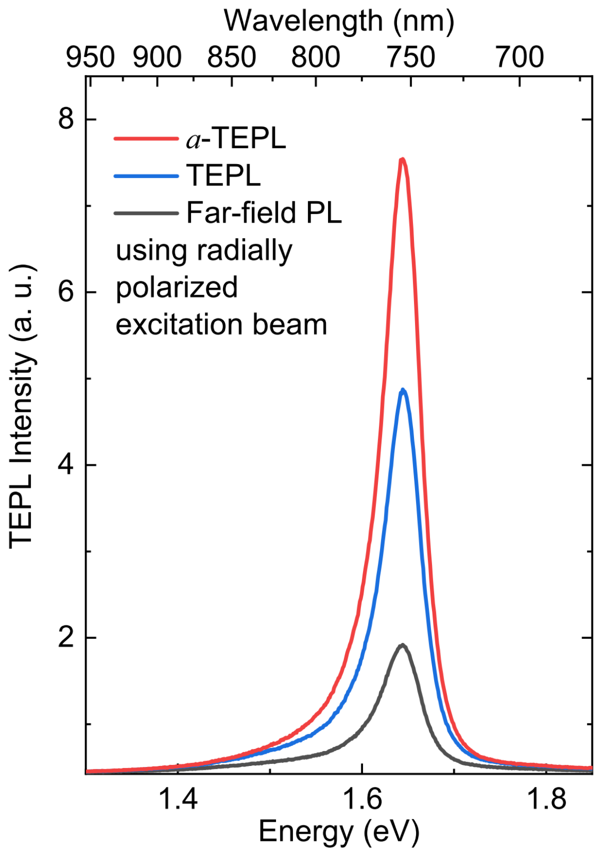

Supplementary Figure 5. Comparison of far-field PL (black) and TEPL spectra of a WSe_2 monolayer without (blue) and with wavefront shaping (*a*-TEPL, red) using a radially polarized excitation beam.

SUPPLEMENTARY NOTE 6. REPRODUCIBILITY TEST OF *A*-TEPL WITH DIFFERENT TIPS

We have tried more than 10 tips and obtained very reproducible enhancement effects for the different tips. TEPL and TERS intensities were generally increased 1.5 $\sim$ 2.5 times with the optimized wavefront for the majority of tips used. We provide more results of *a*-TEPL with different tips as shown in the Supplementary Figure 6 below.

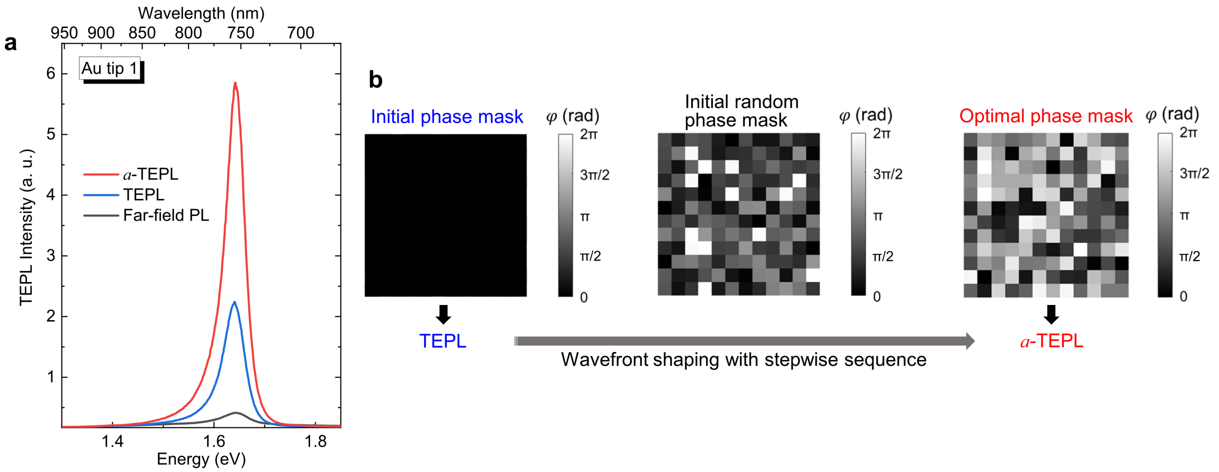

Supplementary Figure 6. Au tip 1: (a) Comparison of far-field PL (black) and TEPL spectra of a WSe_2 monolayer without (blue) and with wavefront shaping (*a*-TEPL, red). (b) Phase masks used for the wavefront shaping of the measurement (a).

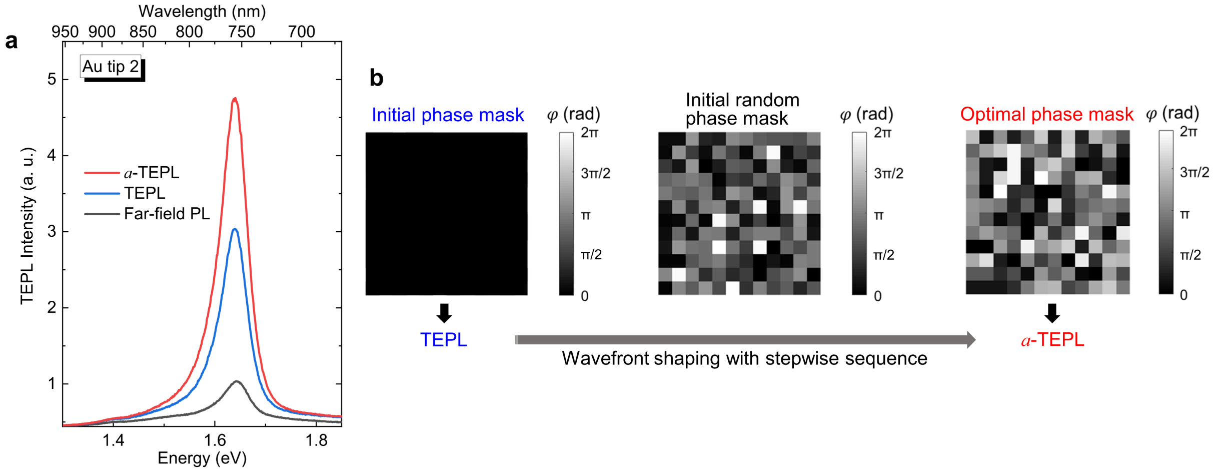

Supplementary Figure 7. Au tip 2: (a) Comparison of far-field PL (black) and TEPL spectra of a WSe_2 monolayer without (blue) and with wavefront shaping (*a*-TEPL, red). (b) Phase masks used for the wavefront shaping of the measurement (a).

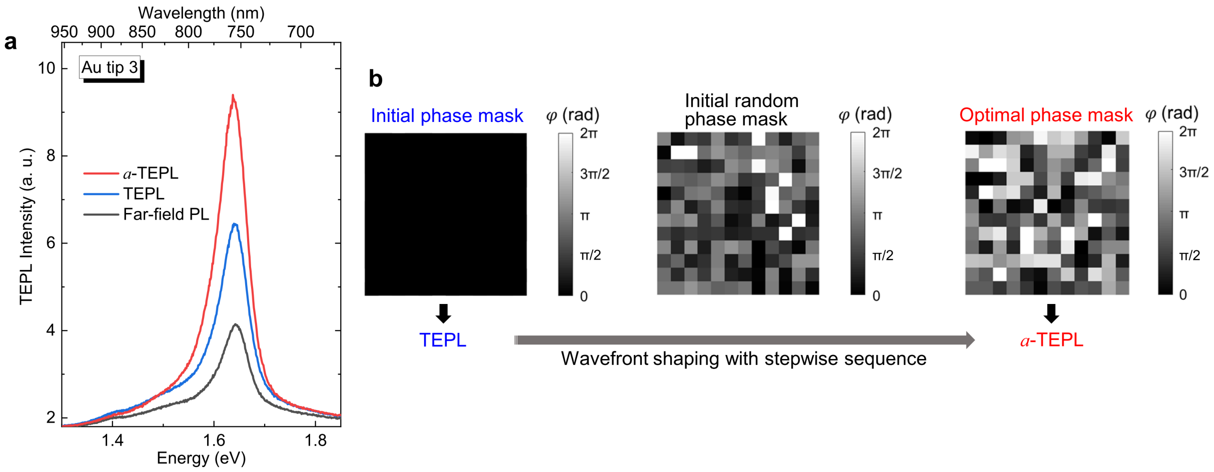

Supplementary Figure 8. Au tip 3: (a) Comparison of far-field PL (black) and TEPL spectra of a WSe_2 monolayer without (blue) and with wavefront shaping (*a*-TEPL, red). (b) Phase masks used for the wavefront shaping of the measurement (a).

SUPPLEMENTARY NOTE 7. OBSERVATION OF IR-ACTIVE MODES WITH TERS

The transitions in vibration levels of molecules induced by optical fields E and optical field gradients ∇E are described by the perturbation Hamiltonian $\mathcal{H}$ given by [16]

$$\mathcal{H} = \Big(\alpha_{\alpha\beta} E_\beta + \frac{1}{3} A_{\alpha,\beta\gamma} \frac{\partial E_{\beta\gamma}}{\partial r}\Big) E_\alpha + \frac{1}{3}\Big(A_{\gamma,\alpha\beta} E_\gamma + C_{\alpha\beta,\gamma\delta} \frac{\partial E_{\gamma\delta}}{\partial r}\Big) \frac{\partial E_{\alpha\beta}}{\partial r} + \cdots \,, \tag{3}$$

where the subscripts $\alpha, \beta, \gamma, \delta$ indicate coordinates in the Cartesian system and $\alpha_{\alpha\beta}$, $A_{\alpha,\beta\gamma}$, $A_{\gamma,\alpha\beta}$, and $C_{\alpha\beta,\gamma\delta}$ are the dipole-dipole, dipole-quadrupole, and quadrupole-quadrupole polarizabilities of a molecule [17]. In far-field vibrational spectroscopy in the visible region, dipole-dipole interactions induced by optical fields result in Raman scattering responses, whereas dipole-quadrupole and quadrupole-quadrupole interactions coupled to optical field gradients result in the activation of IR modes. The IR modes are hard to observe because of much smaller optical field gradient amplitude compared with optical field amplitude. On the other hand, in the tip-enhanced excitation with plasmonic tips, the strong optical field gradients in both in-plane and out-of-plane directions (Fig. 3d) near the tip cannot be ignored. These strong optical field gradients coupled to dipole-quadrupole and quadrupole-quadrupole interactions make possible to observe IR-active modes in TERS measurement [18, 19]. In our TERS experiment with wavefront shaping, we can observe specific IR-active modes with the ability to turn modes on and off, by modulating the tip-SPP polarization dynamically.

SUPPLEMENTARY NOTE 8. DYNAMIC SWITCHING OF IR-ACTIVE MODE WITH *A*-TERS

First of all, we confirm that the observation of the IR-active mode is solely enabled by the wavefront shaping effect in our experiment. Specifically, we verified that i) the IR-active mode was observed at the same spatial location and ii) it can be switched on and off. We could observe the IR-active mode in a specific phase mask and the signal was very robust. To verify the wavefront shaping effect, we swept the phase of a SLM segment with the relative phase difference of $2n\pi$ (n is an integer). As shown in the Supplementary Figure 9 below, we observed the IR-active mode only at the phases of 0, π, 2π, ... $n\pi$, reproducibly. In addition, since we can change the spatial phase of the wavefront with 60 Hz, we can turn it on and off with <20 ms temporal resolution. We believe this experiment clarifies that the IR-active mode was observed by the wavefront shaping.

In contrast to the previous studies observing IR-active modes in the static plasmonic cavities [20–22], our approach provides 'dynamic controllability' to turn IR-active modes on and off through the systematic phase modulation with <20 ms temporal resolution. The static plasmonic cavity, e.g., NPoM, has very small mode volume but it quite unstable in ambient condition. Hence, the IR-active modes can randomly appear owing to the field gradient effect [23]. In contrast, our approach is based on shear-force AFM and we are able to control the tip-sample distance with 0.2 nm precision under ambient conditions [24]. In our *a*-TERS experiment, the tip-sample distance was maintained at $\sim$2 nm to avoid unwanted perturbation effect. Therefore, in addition to tip-enhanced nano-spectroscopy applications, our adaptive near-field approach can be more broadly used to control light-matter interactions in static plasmonic cavities [25–27].

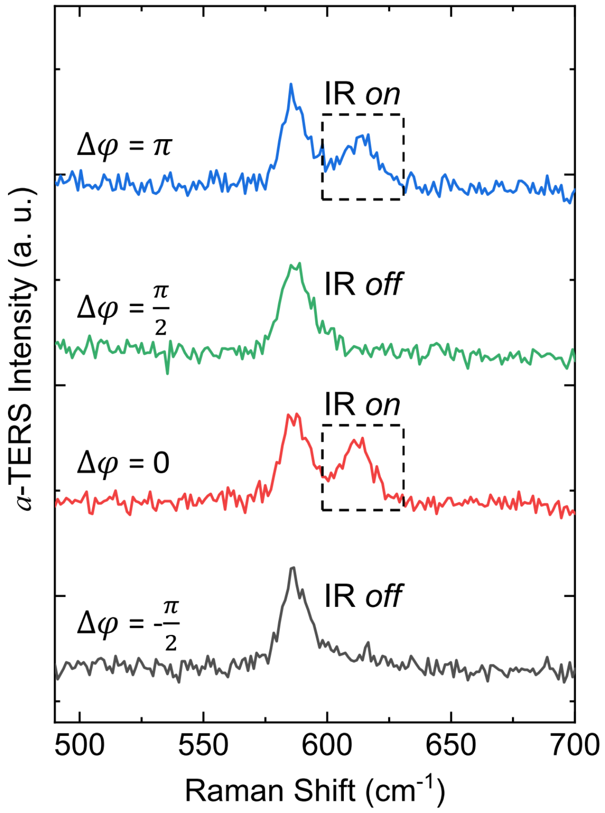

Supplementary Figure 9. Adaptive TERS spectra of BCB molecules with changing the optical phase of a specific segment in a spatial phase mask. The IR-active mode is observed with a period of π, reproducibly.

SUPPLEMENTARY NOTE 9. PROBING CONFORMATIONAL HETEROGENEITY OF BCB MOLECULES WITH *A*-TERS

Fig. 4f in the main text shows *a*-TERS spectra with changing spatial phase of the wavefront. This set of spectra was obtained in a specific time segment during the procedure of stepwise sequencing. The observed spectral fluctuation appeared randomly with changing phase mask. Although we cannot quantitatively analyze the rotational or spectral diffusions as in our previous low temperature work [28] due to the inhomogeneous broadenings at room temperature, we could analyze that the observed TERS peak shifts originate from the different orientations of probing molecules because the near-field polarization at the tip apex can be changed during the wavefront shaping. Inversely, this work demonstrates an important result that probing conformational heterogeneity is feasible at room temperature with *a*-TERS. We provide the control experimental result of *a*-TERS spectra as a function of time at the same location of the sample with the fixed phase mask. As can be seen in Supplementary Figure 10 below, spectral fluctuation is not observed with the fixed wavefront since the stationary near-field polarization selectively probes the molecules with specific orientation. Hence, the result of Fig. 4f shows conformational heterogeneity of molecules.

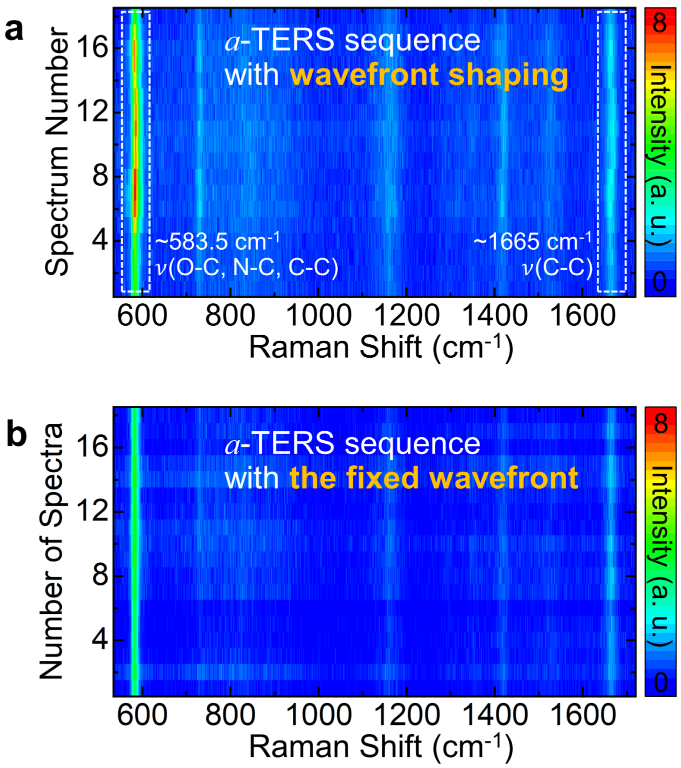

Supplementary Figure 10. Time-series of a-TERS spectra with dynamic wavefront shaping (a) and with the fixed wavefront (b), exhibiting spectral fluctuation through the dynamic wavefront shaping due to the symmetry-selective TERS measurement of heterogeneously oriented molecules.

SUPPLEMENTARY NOTE 10. PL QUENCHING BEHAVIOR OF A TMD MONOLAYER TRANSFERRED ONTO THE GOLD FILM

Since we used the thin gold film (∼10 nm thick) as a substrate for a TMD monolayer, we expect the far-field PL intensity (d > 15 nm) to be decreased due to nonradiative quenching. But, when the Au tip approaches the TMD monolayer with a few nm gap, we expect the suppressed PL quenching because the spontaneous emission is coupled to the antenna mode with its fs-radiative decay [24, 29, 30]. For the far-field PL quenching by the thin gold film, we recently performed control experiment with MoS_2 monolayers. When a MoS_2 monolayer is transferred onto the thin gold film, we expect that there is a thin water layer between them. As shown in the figure below, when the excitation laser power was 3 μW, PL intensity of the TMD crystal was not changed. On the other hand, when we used the high excitation power of >100 μW, the PL intensity was gradually decreased with respect to time up to ∼50 %. From these results, we guess that the water layer was evaporated by the high-power excitation which leads to the increased PL quenching by the reduced distance between the gold film and the TMD crystal.

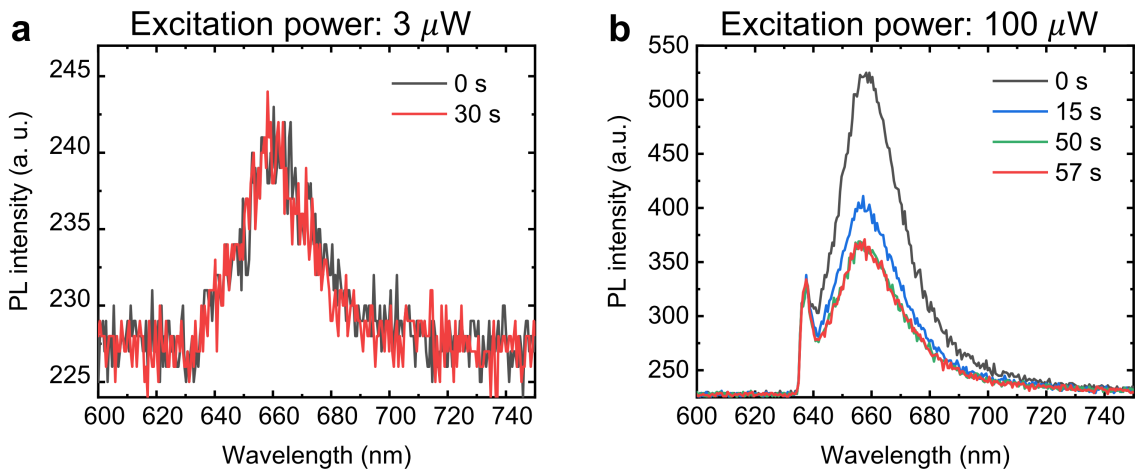

Supplementary Figure 11. (a, b) PL spectra of a MoS_2 monolayer with respect to the excitation time, exhibiting the PL quenching properties at the high excitation power. The CVD-grown MoS_2 monolayer is transferred onto the thin gold film through a wet-transfer method. We assume that there is a water layer between the TMD crystal and the gold film which can suppress the PL quenching phenomenon. When we use an excitation laser power of 3 μW, PL intensity is not changed (a). On the other hand, when use an excitation laser power of $>$100 μW, PL intensity is gradually decreased due to the PL quenching (b). We believe this PL quenching is originated from the evaporated water layer due to the thermal energy of the excitation laser.

[1] Gjonaj, B. *et al.* Focusing and scanning microscopy with propagating surface plasmons. *Phys. Rev. Lett.* **110**, 266804 (2013).

[2] Lee, S.-Y. *et al.* Plasmonic meta-slit: shaping and controlling near-field focus. *Optica* **2**, 6–13 (2015).

[3] Kim, H. *et al.* Synthesis and dynamic switching of surface plasmon vortices with plasmonic vortex lens. *Nano Lett.* **10**, 529–536 (2010).

[4] Gorodetski, Y., Niv, A., Kleiner, V. & Hasman, E. Observation of the spin-based plasmonic effect in nanoscale structures. *Phys. Rev. Lett.* **101**, 043903 (2008).

[5] Zhao, C., Liu, Y., Zhao, Y., Fang, N. & Huang, T. J. A reconfigurable plasmofluidic lens. *Nat. Commun.* **4**, 1–8 (2013).

[6] Noh, H., Chong, Y., Stone, A. D. & Cao, H. Perfect coupling of light to surface plasmons by coherent absorption. *Phys. Rev. Lett.* **108**, 186805 (2012).

[7] Man, Z. *et al.* Dynamic plasmonic beam shaping by vector beams with arbitrary locally linear polarization states. *Appl. Phys. Lett.* **105**, 011110 (2014).

[8] Lin, J. *et al.* Polarization-controlled tunable directional coupling of surface plasmon polaritons. *Science* **340**, 331–334 (2013).

[9] Xu, Q. *et al.* Polarization-controlled asymmetric excitation of surface plasmons. *Optica* **4**, 1044–1051 (2017).

[10] Zhang, X. *et al.* Anomalous surface wave launching by handedness phase control. *Adv. Mater.* **27**, 7123–7129 (2015).

[11] Norrman, A., Ponomarenko, S. A. & Friberg, A. T. Partially coherent surface plasmon polaritons. *EPL (Europhysics Letters)* **116**, 64001 (2017).

[12] Tight focusing induced non-uniform polarization change in reflection for arbitrarily polarized incident light. *Opt. Commun.* **443**, 26 – 33 (2019).

[13] Kazemi-Zanjani, N., Vedraine, S. & Lagugné-Labarthet, F. Localized enhancement of electric field in tip-enhanced raman spectroscopy using radially and linearly polarized light. *Opt. Express* **21**, 25271–25276 (2013).

[14] Stadler, J., Schmid, T. & Zenobi, R. Nanoscale chemical imaging using top-illumination tip-enhanced Raman spectroscopy. *Nano Lett.* **10**, 4514–4520 (2010).

[15] Neacsu, C. C., Berweger, S. & Raschke, M. B. Tip-enhanced Raman imaging and nanospectroscopy: sensitivity, symmetry, and selection rules. *NanoBiotechnol.* **3**, 172–196 (2007).

[16] Buckingham, A. D. Permanent and induced molecular moments and long-range intermolecular forces. *Adv. Chem. Phys.: Intermolecular Forces* 107–142 (1967).

[17] Fang, Y., Zhang, Z., Chen, L. & Sun, M. Near field plasmonic gradient effects on high vacuum tip-enhanced Raman spectroscopy. *Phys. Chem. Chem. Phys.* **17**, 783–794 (2015).

[18] Sun, M., Fang, Y., Zhang, Z. & Xu, H. Activated vibrational modes and fermi resonance in tip-enhanced Raman spectroscopy. *Phys. Rev. E* **87**, 020401 (2013).

[19] Zhang, Z., Sun, M., Ruan, P., Zheng, H. & Xu, H. Electric field gradient quadrupole raman modes observed in plasmon-driven catalytic reactions revealed by HV-TERS. *Nanoscale* **5**, 4151–4155 (2013).

[20] Benz, F. *et al.* Single-molecule optomechanics in "picocavities". *Science* **354**, 726–729 (2016).

[21] Carnegie, C. *et al.* Room-temperature optical picocavities below 1 nm^3 accessing single-atom geometries. *J. Phys. Chem. Lett.* **9**, 7146–7151 (2018).

[22] Shin, H.-H. *et al.* Frequency-domain proof of the existence of atomic-scale SERS hot-spots. *Nano Lett.* **18**, 262–271 (2018).

[23] Meng, L., Yang, Z., Chen, J. & Sun, M. Effect of electric field gradient on sub-nanometer spatial resolution of tip-enhanced Raman spectroscopy. *Sci. Rep.* **5**, 1–5 (2015).

[24] Park, K.-D., Jiang, T., Clark, G., Xu, X. & Raschke, M. B. Radiative control of dark excitons at room temperature by nano-optical antenna-tip Purcell effect. *Nat. Nanotech.* **13**, 59–64 (2018).

[25] Lindquist, N. C., de Albuquerque, C. D. L., Sobral-Filho, R. G., Paci, I. & Brolo, A. G. High-speed imaging of surface-enhanced Raman scattering fluctuations from individual nanoparticles. *Nat. Nanotechnol.* **14**, 981–987 (2019).

[26] de Albuquerque, C. D. L. *et al.* Dynamic imaging of multiple SERS hotspots on single nanoparticles. *ACS Photon.* **7**, 434–443 (2020).

[27] Richard-Lacroix, M. & Deckert, V. Direct molecular-level near-field plasmon and temperature assessment in a single plasmonic hotspot. *Light: Sci. Appl.* **9**, 1–13 (2020).

[28] Park, K.-D. *et al.* Variable-temperature tip-enhanced Raman spectroscopy of single-molecule fluctuations and dynamics. *Nano Lett.* **16**, 479–487 (2016).

[29] Kravtsov, V., Berweger, S., Atkin, J. M. & Raschke, M. B. Control of plasmon emission and dynamics at the transition from classical to quantum coupling. *Nano Lett.* **14**, 5270–5275 (2014).

[30] Kongsuwan, N. *et al.* Suppressed quenching and strong-coupling of purcell-enhanced single-molecule emission in plasmonic nanocavities. *ACS Photon.* **5**, 186–191 (2018).
